# Role of HPV16 E1 in cervical carcinogenesis

**DOI:** 10.3389/fcimb.2022.955847

**Published:** 2022-07-28

**Authors:** Fern Baedyananda, Thanayod Sasivimolrattana, Arkom Chaiwongkot, Shankar Varadarajan, Parvapan Bhattarakosol

**Affiliations:** ^1^Division of Virology, Department of Microbiology, Faculty of Medicine, Chulalongkorn University, Bangkok, Thailand; ^2^Department of Molecular and Clinical Cancer Medicine, Institute of Systems, Molecular and Integrative Biology, University of Liverpool, Liverpool, United Kingdom; ^3^Medical Microbiology Interdisciplinary Program, Graduate School, Chulalongkorn University, Bangkok, Thailand; ^4^Center of Excellence in Applied Medical Virology, Department of Microbiology, Faculty of Medicine, Chulalongkorn University, Bangkok, Thailand

**Keywords:** HPV, HPV16, HPV16 E1, cervical carcinogenesis, cervical cancer

## Abstract

Cervical cancer is the fourth most common cancer in women worldwide. More than 90% of cases are caused by the human papillomavirus (HPV). Vaccines developed only guard against a few HPV types and do not protect people who have already been infected. HPV is a small DNA virus that infects the basal layer of the stratified epithelium of the skin and mucosa through small breaks and replicates as the cells differentiate. The mucosal types of HPV can be classified into low-risk and high-risk groups, based on their association with cancer. Among HPV types in high-risk group, HPV type 16 (HPV-16) is the most common, causing 50% of all cancer cases. HPV infection can occur as transient or persistent infections, based on the ability of immune system to clear the virus. Persistent infection is characterized by the integration of HPV genome. HPV-16 exhibits a different integration pattern, with only 50% reported to be integrated at the carcinoma stage. Replication of the HPV genome depends on protein E1, an ATP-dependent helicase. E1 is essential for the amplification of the viral episome in infected cells. Previous studies have shown that E1 does not only act as a helicase protein but is also involved in recruiting and interacting with other host proteins. E1 has also been deemed to drive host cell proliferation. Recent studies have emphasized the emerging role of HPV E1 in cervical carcinogenesis. In this review, a possible mechanism by which E1 drives cell proliferation and oncogenesis will be discussed.

## Introduction

Cervical cancer (CC) is one of the most frequent common malignancies in women worldwide. Persistent infection with some types of human papillomaviruses (HPVs) is the major factor contributing to the development of cervical carcinogenesis (Schiffman et al., 2007; Petry, 2014). To observe the histological abnormality of the patients, colposcopy with biopsy, endocervical scraping, and cone biopsies are performed. The abnormalities detected in biopsy samples are termed cervical intraepithelial neoplasia (CIN) or dysplasia. These CINs are divided into 3 major stages, i.e., CIN1, CIN2, and CIN3, including carcinoma *in situ* (Waxman et al., 2012). Moreover, if cancerous cells are observed, they will be identified as squamous cell carcinoma (SCC) or adenocarcinoma.

HPVs are epitheliotropic viruses with an 8 kbp double-stranded circular DNA genome contained in the naked icosahedral capsid. The HPV’s genomic DNA is packaged as a minichromosome with cellular nucleosomal histone (Acheson, 2011). HPV belongs to family *Papillomaviridae* and currently divided into 5 genera, namely *Alpha-*, *Beta*-, *Gamma*-, *Mupa*- and *Nupapapillomavirus* containing members, all of which can infect humans (Brooks et al., 2013). To this date, more than 200 HPV types have been identified (McBride, 2017). In the *Alphapapillomavirus* genus, HPVs are divided into two major groups - high-risk HPVs (Hr-HPVs) and low-risk HPVs (Lr-HPVs) - based on the cancer risk associated with their infection. Hr-HPVs include types 16, 18, 31 and 33 (Ghittoni et al., 2015), whereas Lr-HPVs include types 6 and11 (Brooks et al., 2013).

Papillomaviruses can infect a wide range of animal species; however, each type of papillomavirus is highly host- and tissue-specific. HPV infects cells in the basal layer of the epithelium, following wounds or breaks in the epithelium. Upon infection, HPVs maintain their genome as an extrachromosomal element, or episome, in the nucleus of infected cells. At this infection stage, and while the cells are in the lower strata of the epithelium, only the early genes are expressed (Graham, 2017). When the infected cell proliferates, the HPV genome replicates and increases the episomal copy numbers in the cell. The viral genome is replicated along with host cell DNA replication; after cell division occurs, the daughter cell contains a copy of the HPV genome. There is no new virion progeny produced in this initial phase. When the cell proliferates and differentiates, HPV DNA replication increases, resulting in a high episomal viral genome copy number (Burd, 2003). Finally, in the upper strata of the epithelium, the late proteins are encoded for capsid formation and released in the upper strata of the epithelium (Longworth and Laimins, 2004). Most HPV infections are transient and are cleared within approximately 2 years; nevertheless, if the host immune system is unable to clear the infection, a persistent infection occurs, possibly leading to viral genome integration into the host cell (Plummer et al., 2007).

The HPV genome encodes about 8 open-reading frames (ORFs), of which are divided into three functional regions - early (E) region, late (L) region, and noncoding part or long control region (LCR) (Yousefi et al., 2021). The early region encodes 6 early proteins (E1, E2, E4, E5, E6, and E7), whereas the late region encodes only 2 structural proteins, L1, and L2, that compose the capsid of HPVs (Chan et al., 2019). Whilst E1 has been shown to encode the primary protein responsible for viral replication (Berg and Stenlund, 1997), E2 is involved in transcriptional regulation (Chojnacki and Melendy, 2018), and E4 regulates virion release (Doorbar et al., 1996). The late proteins L1 and L2 are the major (80%) and minor (20%) capsid proteins, respectively. In contrast to these proteins, the functional roles of E5, E6 and E7 have been extensively characterized in the context of cancer. For instance, HPV E5 plays a role in immune evasion whereas E6 and E7 causes hyper-proliferation of the cell and cancer progression (Crook et al., 1991; Ganguly, 2012). The major role of the HPV E6 oncoprotein is to immortalize the cells *via* the ubiquitin-dependent proteasome degradation of p53, a cellular tumor suppressor protein, thus evading cancer cell death (Nguyen et al., 2002; Kelley et al., 2005). In addition, E6 can also degrade other apoptotic signaling cascade molecules (Filippova et al., 2002). HPV E7 oncoprotein plays a critical role in cervical carcinogenesis through dysregulation of cell cycle. This protein inactivates the retinoblastoma tumor suppressor protein (pRb) and downregulates E2F (Moody and Laimins, 2010). It is therefore evident that the role of HPV proteins, other than E5, E6, and E7, in carcinogenesis is under-investigated. Recently, it has been shown that HPV E1 may be involved in carcinogenesis. Here, we will review the possible role of E1 in cervical carcinogenesis.

## The structure of E1 protein

**Figure 1:**
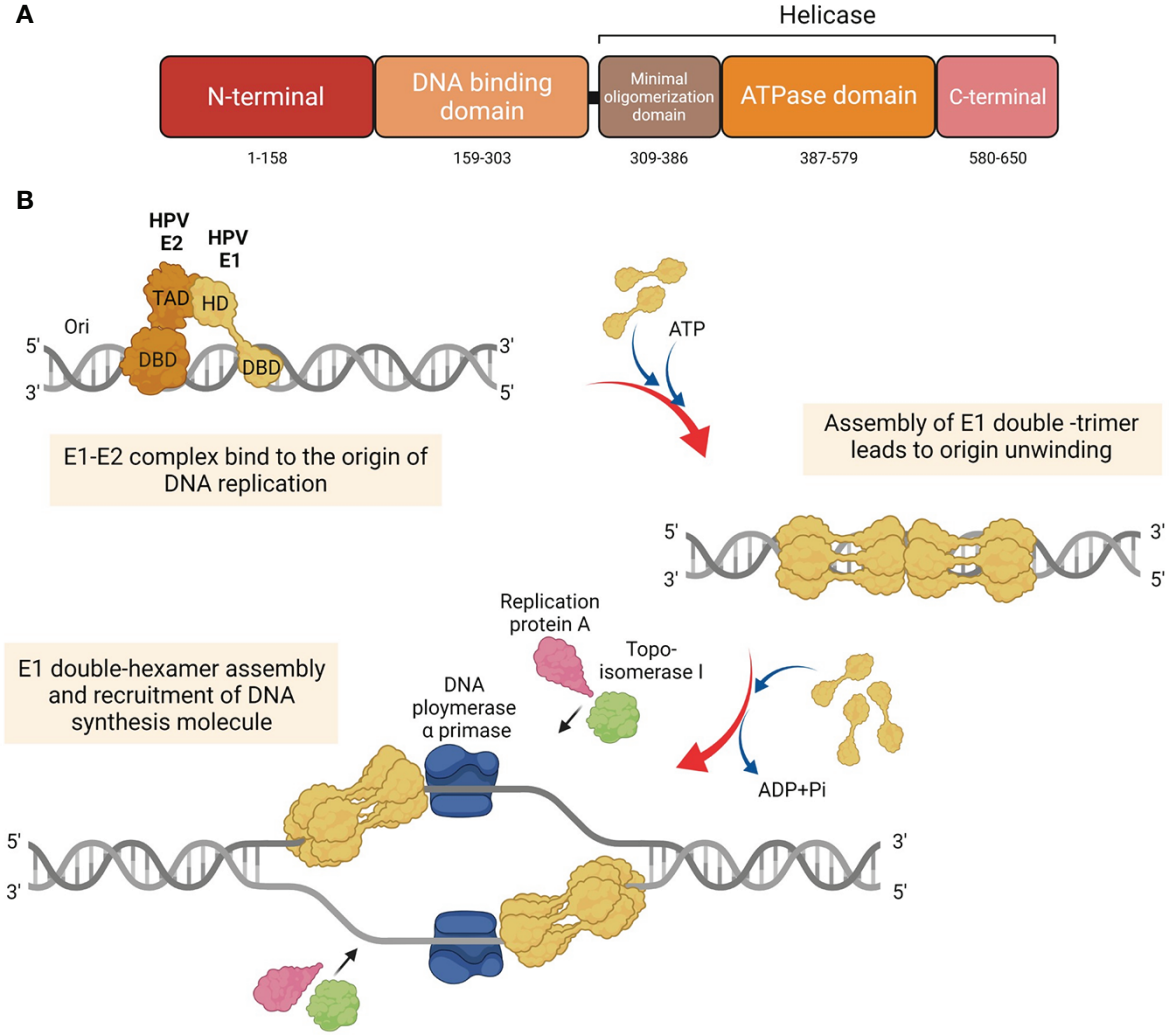
Structure and function of HPV E1. **(A)** Diagram of the HPV E1 protein domains. Three major functional domains of HPV E1, i.e., N-terminal, DNA binding domain (DBD), and the domain which construct the helicase domain: minimal oligomerization domain, ATPase domain, and C-terminal brace. **(B)** Schematic representation of the initiation of the HPV DNA replication associated with E1 protein. HPV E1 proteins are recruited to bind to the E1 DNA binding site at the origin by HPV E2. E1 and E2 are assemble as E1-E2-ori ternary complex. Additional of E1 proteins are recruited to assemble as E1 double-trimer and double-hexamer, respectively. Then, the ds-DNA are unwound, and the DNA replication are initiated by the recruitment of host DNA replication factors.

The E1 protein serves as the primary replication protein of HPV and is an ATP-dependent helicase that binds to the viral origin of replication and unwinds the viral DNA to initiate replication (Hughes and Romanos, 1993). Across all papillomaviruses, the E1 protein is the most conserved, primarily owing to its helicase function, which is essential for the viral episome replication. It is possible that all phases of the viral replication cycle namely, establishment, maintenance, and amplification, require E1 function. The E1 protein consists of three main domains, each with a distinct and important function, i.e., The N-terminal regulatory domain, the DNA binding domain, and the helicase domain (**Figure 1A**) (Bergvall et al., 2013). The N-terminal of the protein contains the nuclear localization signal and the nuclear export signal, which functions to transport E1 between the nucleus and cytoplasm. The DNA binding domain recognizes specific sequences near the viral origin of replication, which is bound by the helicase domain to form a doughnut shaped complex around viral DNA (Enemark and Joshua-Tor, 2006).

## Mechanisms of HPV E1 in viral DNA replication

To initiate HPV DNA replication, E1 interacts with E2 (Berg and Stenlund, 1997; Bergvall et al., 2013; Porter et al., 2017), which aids in its recruitment to the origin of replication (Frattini and Laimins, 1994) (**Figure 1B**). It must be noted that E1 can also establish viral replication in an E2 independent manner, albeit with lower efficiency (Bonne-Andrea et al., 1997). Upon binding to the origin of replication as an E1-E2-ori ternary complex, additional E1 molecules are recruited to assemble E1 double-trimers and E1 double-hexamers, which whilst unwinding DNA also recruits several DNA replication factors, such as DNA polymerase α primase (Pol α-prim), topoisomerase I (Topo I), and replication protein A (RPA) (Loo and Melendy, 2004; Bergvall et al., 2013).

## Relationship between HPV16 E1 expression and cervical cancer progression

The most well-characterized function of HPV in cervical carcinogenesis is the overexpression of E6 and E7 oncoproteins, which mainly target p53 and pRb tumor suppressor proteins (Munger et al., 1992; Mantovani and Banks, 2001). However, a relationship between HPV E1 expression and cervical cancer progression has been reported. Interestingly, HPV16 E1 mRNA expression is positively correlated with cervical cancer progression (Baedyananda et al., 2017). This finding was in agreement with another independent study that reported lower E1 expression in patients with low-grade squamous intraepithelial lesion (LSIL) compared to high-grade squamous intraepithelial lesion (HSIL) and cancer (Schmitt et al., 2011).

The physical state of the HPV genome, namely integrated, episomal, or mixed, is correlated with cervical carcinogenesis (Pirami et al., 1997; Williams et al., 2011). HPV integration is associated with overexpression of E6 and E7 (Jeon et al., 1995), which in turn have been shown to enhance the pathogenicity of HPV (Munger et al., 1992; Burke et al., 2012; Burke et al., 2014). However, recent findings have reported that E1 mRNA expression is neither impacted by the number of copies of the HPV16 genome, nor its physical state (Baedyananda et al., 2017). Similar observations have also been made in relation to E6 and E7 (Wang-Johanning et al., 2002), which implicates other factors, including epigenetic modifications in the control of HPV mRNA expression. Moreover, HPV genome has been reported to be highly methylated in cervical cancer samples with high copy numbers of integrated HPV (Chaiwongkot et al., 2013). In agreement, E1 promoters p97 and p670 are hypermethylated in cervical cancer samples when compared to normal samples (Baedyananda et al., 2017), thereby suggesting the possibility of these samples possessing high numbers of HPV genome copies.

## The possible functions of HPV16 E1 in cervical carcinogenesis

### HPV16 E1 dysregulates the expression of genes involved in cell survival

Failure to induce apoptosis could drive tumorigenesis through multiple mechanisms (Ichim and Tait, 2016). In HPV16 E1 overexpressing cells, several host genes that are involved in protein synthesis (RPL36A), metabolism (ALDOC), immune response (ISG20), DNA damage (ATR, BRCA1, and CHK1), and cell proliferation (CREB5, HIF1A, NFKB1, PIK3CA, JMJDIC, TSC22D3, FOXO3), have been shown to be significantly downregulated (Baedyananda et al., 2021). Of the transcriptional factors, CREB5, HIF1A, NFKB1, and PIK3CA, downregulation of NFKB1 and PIK3CA supports tumor growth and survival (Xia et al., 2014; Masoud and Li, 2015). JMJD1C (Jumonji Domain Containing 1C) is a histone demethylase, tumor suppressor protein that is usually found to be reduced or lost in breast cancer (Watanabe et al., 2013). TSC22D3 is a gene that encodes Glucocorticoid-induced leucine zipper (GILZ), which in turn activates FOXO3a-mediated transcription of the pro-apoptotic protein, Bim (Joha et al., 2012). Loss of FOXO3a activity has previously been linked to disease progression in carcinogen-induced lung adenocarcinoma (Blake et al., 2010), neck cancer (Shou et al., 2012) and urothelial cancer (Shiota et al., 2010). How HPV16 E1 promotes cell survival *via* regulation of FOXO3 in cervical carcinogenesis is still unclear.

### HPV16 E1 mutations associated with cervical cancer

A variety of mutations in E1/E2 genes have been identified in cervical cancer samples. The clinical stage of patients with the discovered HPV E1 mutations have also been noted in various studies. Many of these variants were linked with lower grade lesions such as noted in previous studies where a 63-bp duplication variant was associated with lower disease progression (Sabol et al., 2008; Baedyananda et al., 2017). Other mutations have demonstrated reduced abilities to support HPV replication (Yao et al., 2019) and also failed to suppress the viral early promoter (Yao et al., 2019; Hirose et al., 2020). When examining within-host variations of HPV16 it was noted that non-synonymous substitutions occurred more frequently in invasive carcinoma specimens. These mutations also occurred more frequently in E1/E2 regions than in other regions of the viral genome (Hirose et al., 2020). Many factors determine the functional impact of mutations i.e., type of mutation, location of the mutation. Further studies are required to understand how information gained from this could be therapeutically exploited.

### Helicase function of HPV E1 and possible roles in cancer

Genome instability, a hallmark of cancer (Hanahan and Weinberg, 2000; Hanahan and Weinberg, 2011), is caused by several factors that induce DNA damage (Langie et al., 2015), which could be exacerbated by defective DNA damage repair response and tumor suppressor molecules (Negrini et al., 2010). Inactivating mutations in DNA helicases have detrimental effects, such as in Werner syndrome, characterized by the appearance of premature aging features and early onset of age-related diseases such as cardiovascular diseases, diabetes mellitus, and carcinoma (Martin, 1985). Patients with Bloom’s syndrome are also predisposed to carcinogenesis (German, 1997). In addition, cells transformed by the Epstein-Barr virus (EBV) and simian virus 40 (SV40) have exhibited upregulated helicases expression. In the context of HPV infection, E1 helicase facilitates HPV genome replication, *via* the host cells’ DNA damage repair pathways and contributes to carcinogenesis (Moody and Laimins, 2009; King et al., 2010; Fradet-Turcotte et al., 2011; Sakakibara et al., 2011). HPV E1 proteins are able to directly cause double strand DNA breaks in the host genome. The ATR-dependent DNA damage response pathway is activated by HPV replication or presence of HPV replication proteins, E1 and E2. This has been shown by the accumulation of γH2AX, ATR-interacting protein (ATRIP), and topoisomerase IIβ-binding protein 1 (TopBP1) in replication centers. Conversely, the viral oncoproteins E6 and E7 did not play a role in the accumulation of γH2AX, ATRIP and TopBP1 (Reinson et al., 2013).

Moreover, the homeobox transcription factor HOXC13 has been shown to upregulate the expression of HPV16/18 E1 and E2 (Ishii et al., 2020), which may enhance the helicase activity of E1 to result in DNA damage and genome instability. HPV E1’s ability to directly cause host DNA damage and the implications on carcinogenesis should be further explored to better define the role of HPV E1 in carcinogenesis.

### Role of HPV16 E1 in antiviral immune evasion

Type I interferons (IFN), such as, IFN-α and IFN-β, form integral components of the innate immune response, playing important roles in antiviral, anti-proliferative and immunomodulatory functions (Seth et al., 2006). HPV18 E1 modulates the expression of the genes involved in toll-like receptor, interferons and apoptosis pathways, and antiviral interferon-stimulated gene set (Castillo et al., 2014). Similarly, HPV16/18 E1 protein has been shown to enhance the expression of immune response genes, i.e., IFNβ1, IFNλ1, and interferon-stimulated gene (ISG) (Castro-Muñoz et al., 2019), thus implicating roles for HPV E1 in immune modulation, as evasion of the host immune response is key to persistent infection and HPV-related carcinogenesis.

## Conclusion

**Figure 2:**
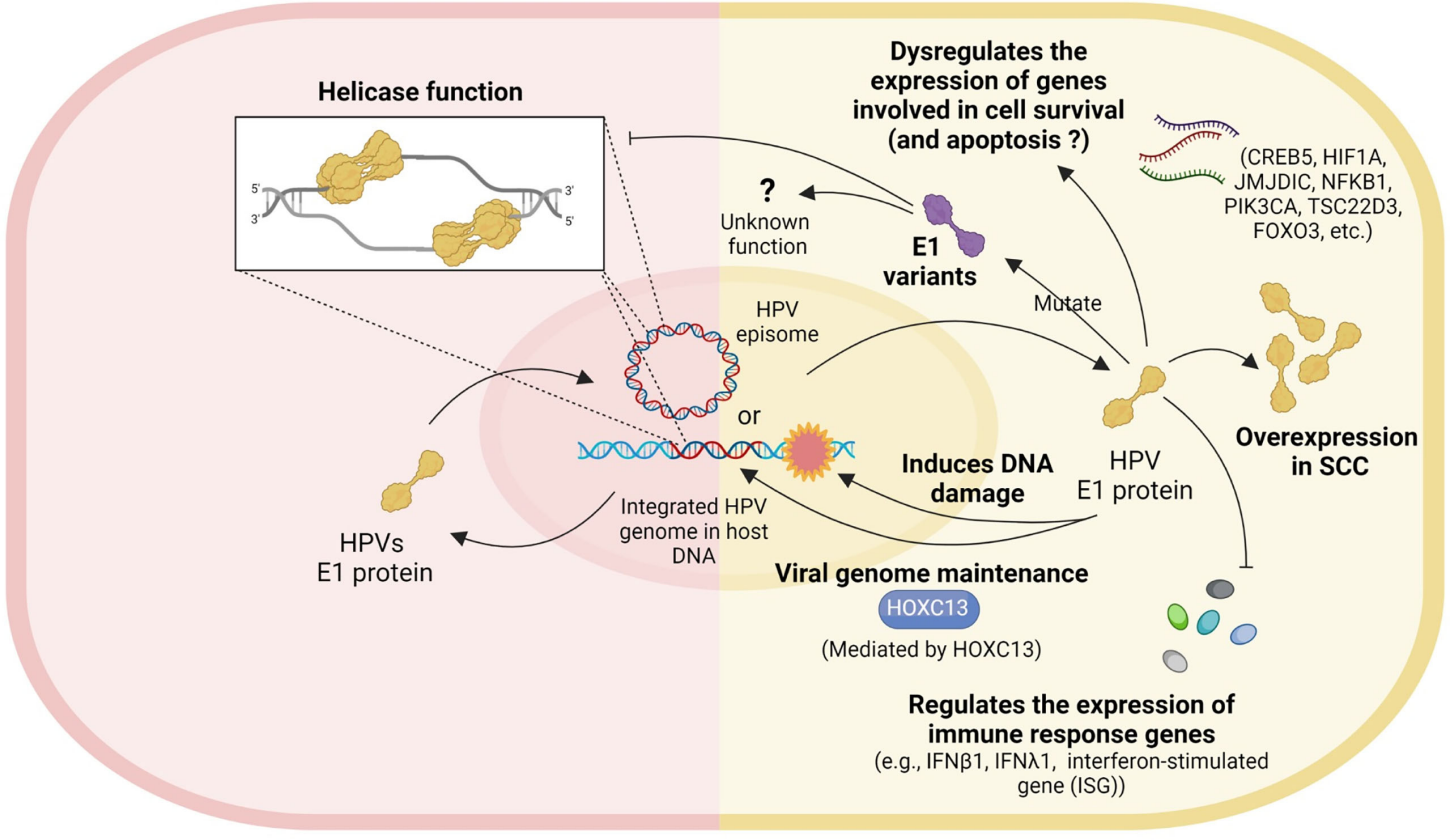
Role of HPV E1. Left panel: the well-known helicase function of HPV. Right panel: the possible roles of HPV E1 in cervical carcinogenesis include dysregulating the expression of genes involved in cell survival, E1 mutation, E1 overexpression, inducing DNA damage, viral genome maintenance, and regulating the expression of immune response genes.

Several studies have explored possible roles of HPV16 E1 in cervical carcinogenesis and have attributed numerous cellular mechanisms, including increased expression of genes involved in cell survival and apoptosis, inhibition of the antiviral immune response, viral genome maintenance, helicase activity and inactivating mutations (**Figure 2**). However, there are no functional evidence of HPV16 E1 directly on cancer development. It is possible that E1 is the only protein with both DNA helicase and ATPase activity, which may or may not be relevant for carcinogenesis. Unfortunately, only few studies worked on HPV16 E1 related to cancer development. For further study, the function of E1 protein in cervical carcinogenesis should be deeply explored. More functional assays related to the hallmarks of cancer, e.g., cell proliferation, apoptosis, cell cycle arrested, migration/invasion, wound healing, and colony formation, should be done in E1 overexpressed/knockdown/knockout cells. Moreover, many genes such as BCL2L1, CSP2, FOXO3a, JMJDIC, and TSC22D3, were dysregulated in either HPV18 or HPV16 E1 overexpressed/knockdown cells (Castillo et al., 2014; Baedyananda et al., 2021). To better understand the E1 associated cellular pathways, the relationship between E1 and those genes as well as E1 protein-protein interaction should be investigated. Since HPV E1 proteins are helicases, studying cells over-expressing HPV16 E1 and observing the impact on genome instability could expand our understanding of the carcinogenic role of HPV16 E1. In addition, the association between the expression of some cellular genes mentioned above and various stage of cervical specimens should be determined to confirm the phenomenon truly occur in human. Taken together, uncovering the cellular and molecular mechanisms underlying HPV16 E1-mediated cervical carcinogenesis is therefore critical to identify novel biomarkers and/or druggable targets and/or a possible forth HPV oncoprotein beside E5, E6 and E7.

## Author contributions

Conceptualization, FB, TS, and PB; Writing – Original Draft Preparation, FB, and TS; Writing – Review & Editing, PB, SV, and AC; Supervision, PB; Project Administration, PB and SV; Funding Acquisition, FB, TS, PB, SV and AC; Figure Illustration, TS. All authors contributed to the article and approved the submitted version.

## Funding

The 100th Anniversary Chulalongkorn University Fund for Doctoral Scholarship, The Overseas Research Experience Scholarship for Graduate Student, Rachadaphiseksomphot Endowment Fund, and Thailand Science Research and Innovation Fund (TSRI), Chulalongkorn University, Bangkok 10330, Thailand and University of Liverpool, UK.

## Acknowledgments

We would like to acknowledge that all figures were created with BioRender.com platform.

## Conflict of interest

The authors declare that the research was conducted in the absence of any commercial or financial relationships that could be construed as a potential conflict of interest.

## Publisher’s note

All claims expressed in this article are solely those of the authors and do not necessarily represent those of their affiliated organizations, or those of the publisher, the editors and the reviewers. Any product that may be evaluated in this article, or claim that may be made by its manufacturer, is not guaranteed or endorsed by the publisher.
